# Neuroprotective Effect of Valproic Acid on Salicylate-Induced Tinnitus

**DOI:** 10.3390/ijms23010023

**Published:** 2021-12-21

**Authors:** Anji Song, Gwang-Won Cho, Karthikeyan A. Vijayakumar, Changjong Moon, Mary Jasmin Ang, Jahae Kim, Ilyong Park, Chul Ho Jang

**Affiliations:** 1 Department of Biology, College of Natural Science, Chosun University, Gwangju 61452, Korea; dkswl511@naver.com (A.S.); gwcho@chosun.ac.kr (G.-W.C.); cartkn1991@gmail.com (K.A.V.); 2 BK21 FOUR Education Research Group for Age-Associated Disorder Control Technology, Department of Integrative Biological Science, Chosun University, Gwangju 61452, Korea; 3 Department of Veterinary Anatomy, College of Veterinary Medicine and BK21 FOUR Program, Chonnam National University, Gwangju 61186, Korea; ang.maryjasmin@gmail.com; 4 Department of Nuclear Medicine, Chonnam National University Hospital, Gwangju 61469, Korea; jhbt0607@hanmail.net; 5 Department of Biomedical Engineering, School of Medicine, Dankook University, Cheonan 31116, Korea; piyong@dankook.ac.kr; 6 Department of Otolaryngology, Chonnam National University Medical School, Gwangju 61469, Korea

**Keywords:** auditory dysfunction, tinnitus, salicylate, valproic acid, glutamate excitotoxicity, ROS, apoptosis

## Abstract

High-dose salicylate induces temporary moderate hearing loss and the perception of a high-pitched tinnitus in humans and animals. Previous studies demonstrated that high doses of salicylate increase N-methyl-d-aspartate (NMDA) receptor levels, resulting in a rise in Ca^2+^ influx and induction of excitotoxicity. Glutamate excitotoxicity is associated with failure in the maintenance of calcium homeostasis, mitochondrial dysfunction, and production of reactive oxygen species (ROS). Valproic acid (VPA) is widely used for the management of bipolar disorder, epilepsy, and migraine headaches, and is known to regulate NMDA receptor activity. In this study, we examined the beneficial effects of VPA in a salicylate-induced tinnitus model in vitro and in vivo. Cells were pretreated with VPA followed by salicylate treatment. The expression levels of NMDA receptor subunit NR2B, phosphorylated cAMP response element-binding protein—an apoptosis marker, and intracellular levels of ROS were measured using several biochemical techniques. We observed increased expression of NR2B and its related genes *TNFα* and *ARC*, increased intracellular ROS levels, and induced expression of cleaved caspase-3. These salicylate-induced changes were attenuated in the neuronal cell line SH-SY5Y and rat cortical neurons after VPA pretreatment. Together, these results provide evidence of the beneficial effects of VPA in a salicylate-induced temporary hearing loss and tinnitus model.

## 1. Introduction

Chronic tinnitus is an auditory phantom perception; it is defined not as a disease but as a prevalent symptom. According to epidemiological survey reports, the prevalence of tinnitus is approximately 10%–15% in the adult population. According to a study, severe tinnitus affected 1%–2% of adults and, in 0.5% of adults, it disrupted the ability to live a normal life [1,2]. Additionally, the majority of sensorineural hearing loss, such as noise-induced hearing loss or presbycusis, is accompanied by tinnitus [3]. 

Among the several treatment options, such as noise suppression using a sound generator or tinnitus retraining therapy, medication has been consistently recommended by physicians. Although no specific drugs have been approved by the FDA to treat tinnitus, drugs are still available to reduce the severity of the symptoms. To date, frequently prescribed medications are divided into several groups based on their mode of action, such as benzodiazepines, antidepressants, anticonvulsants, and N-methyl-d-aspartate (NMDA) receptor antagonists. 

Recently, anticonvulsants have been increasingly examined for their potential in treating diseases other than epilepsy [4]. They are also being tested for treating tinnitus, especially carbamazepine, which revealed significant benefits for typewriter tinnitus and ear-clicking tinnitus [5,6,7]. Valproic acid (VPA) is another commonly prescribed antiepileptic; however, only one case report has reported its usefulness in tinnitus [8]. Moreover, basic research on the use of VPA for tinnitus has not yet been reported. A tinnitus rat model can be induced by salicylate, which stimulates NMDA receptors [9,10]. Recent studies showed significantly elevated expression of NR2B, which is a subunit of NMDA receptors, and phospho-c-AMP response element-binding protein (p-CREB) in the auditory cortex in a salicylate-induced tinnitus model [9,11,12,13]. 

The aim of the present study was to investigate the effects of VPA on the expression of *ARC*, which is a neuronal immediate early gene, *TNFα*, and NR2B gene and protein, as well as on the levels of p-CREB in the SH-SY5Y cell line and rat cortical neurons. For the in vivo study, we used gap-prepulse inhibition of the acoustic startle reflex (GPIAS) and measured the auditory brainstem level (electrophysiological recordings of auditory brainstem responses; ABR). Furthermore, we examined NR2B expression in the auditory cortex to evaluate whether VPA could reduce salicylate-induced behavioral disturbances as well as the expression of NR2B. Additionally, we evaluated regional ^18^F-2-fluoro-2-deoxy-d-glucose (^18^F-FDG) uptake in the auditory cortex using micro-positron emission tomography (PET) imaging. 

## 2. Results

### 2.1. Gene Expression Levels of ARC, TNFα, and NR2B 

**Figure 1:**
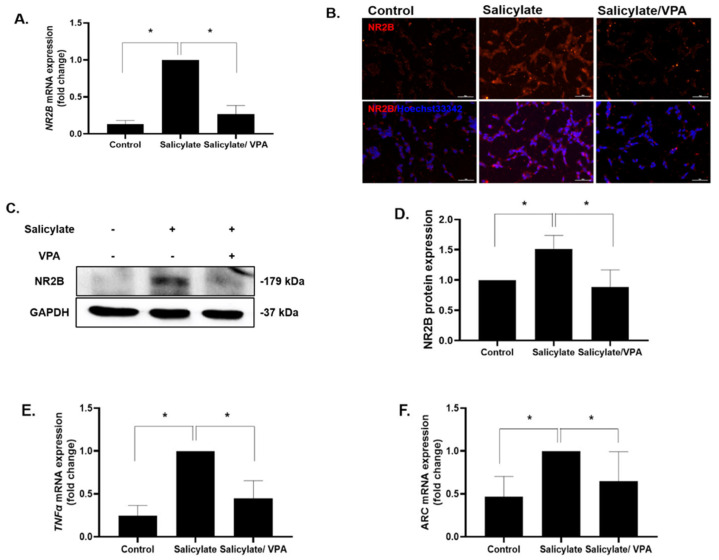
VPA reduces the expression of NR2B and related genes in neuronal SH-SY5Y cell line (**A**) NR2B expression was increased in salicylate treated cells and significantly decreased in VPA-pretreated neuronal differentiated SH-SY5Y cells. Results were determined by quantitative PCR * *p* < 0.05, mean ± SD, *n* = 4), immunocytochemical staining (**B**), and immunoblot analysis ((**C**,**D**); * *p* < 0.05, mean ± SD, *n* = 3). Quantitative PCR results of the inflammatory cytokine TNFα and immediate early gene *ARC* showed changes consistent with NR2B ((**E**,**F**); * *p* < 0.05, mean ± SD, *n* = 6) One-way ANOVA followed by post hoc Tukey test using GraphPad Prism 8.0.

NR2B expression was increased by salicylate treatment and was significantly decreased upon pretreatment with VPA according to the results of qPCR, immunocytochemical staining, and immunoblotting (Figure 1A–D). Immunoblot analysis of NR2B was repeated, and data were quantified (Figure 1D). The increased expression of the inflammatory cytokine TNFα and immediate early gene ARC was reduced when salicylate-treated SH-SY5Y cells were stimulated with VPA (Figure 1E,F). 

### 2.2. Expression of p-CREB 

**Figure 2:**
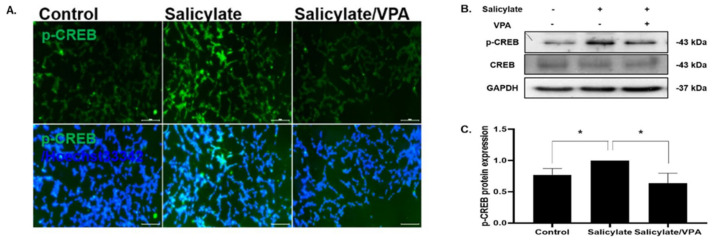
VPA treatment reduces CREB phosphorylation in salicylate-treated SH-SY5Y cells (**A**,**B**). The levels of phosphorylated CREB (p-CREB) were increased in salicylate-treated SH-SY5Y cells and significantly decreased in VPA-pretreated cells. (**C**). Protein expression was quantified using Image J software and normalized with that of CREB and GAPDH (One-way ANOVA followed by post hoc Tukey test using GraphPad Prism 8.0. * *p* < 0.05, mean ± SD, *n* = 3).

The phosphorylation levels of CREB were increased in salicylate-treated cells and then significantly decreased in VPA-pretreated cells, whereas the levels of CREB and the internal standard GAPDH showed no changes upon any treatment (Figure 2A,B). The experiments were repeated, and significant differences were observed between the salicylate group and salicylate/VPA group of cells (Figure 2C).

### 2.3. Neuroprotective Effect via Modulation of ROS Production

**Figure 3:**
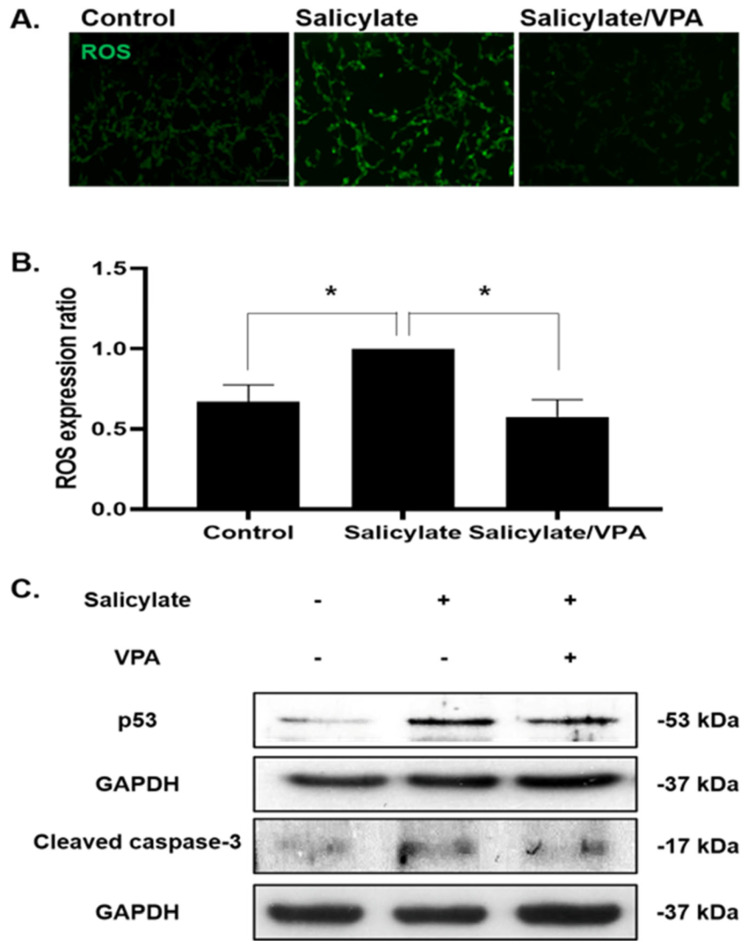
VPA prevents oxidative damage in salicylate-treated cells (**A**) Intracellular ROS levels were increased in salicylate-treated SH-SY5Y cells and significantly decreased in VPA-pretreated cells, as observed by immunocytochemical staining (scale bar = 100 µm). (**B**) ROS levels were quantified using Image J software and are presented in the graph (* *p* < 0.05, mean ± SD, *n* = 4). (**C**) Immunobloting was performed using antibodies against p53 and cleaved caspase-3. The expression levels of the tested proteins were decreased by VPA pretreatment. GAPDH was used as an internal standard.

ROS levels were increased in salicylate-treated cells and were then subsequently reduced following VPA pretreatment (Figure 3A,B). To examine the cell-protective effects of VPA, immunoblot analyses were performed using antibodies against p53 and cleaved caspase-3, demonstrating the fact that pretreatment with VPA prevented apoptosis (Figure 3C). 

### 2.4. Effects of VPA in Rat Cortical Neurons

**Figure 4:**
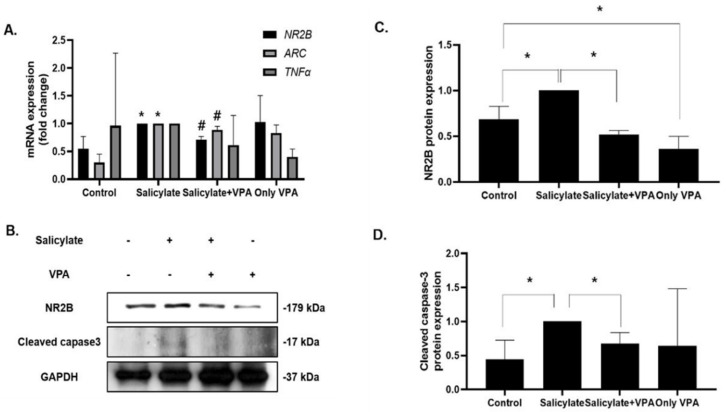
Effect of VPA in rat cortical neurons (**A**) The gene expression levels of NR2B, TNFα, and ARC were increased with salicylate treatment and decreased in VPA-treated rat cortical neurons (* *p* < 0.05, # *p* < 0.05, mean ± SD, *n* = 3). (**B**) The protein expression levels of NR2B ((**C**); * *p* < 0.05, mean ± SD, *n* = 3) and cleaved caspase-3 ((**D**); * *p* < 0.05, mean ± SD, *n* = 4) were measured using immunoblot analysis, quantified using the Image J software, and normalized with those of GAPDH. One-way ANOVA followed by post hoc Tukey test using GraphPad Prism 8.0.

We demonstrated that salicylate stimulated NR2B expression and increased intracellular ROS levels (Figure 2 and Figure 4). VPA pretreatment prevented these harmful effects in SH-SY5Y cells. These results were verified in rat cortical neurons. NR2B expression levels were increased in salicylate-treated cortical neurons and subsequently decreased in VPA-pretreated neurons, as measured by qPCR and immunoblot analysis (Figure 4A–C). Consistent results were obtained from qPCR analysis for ARC and TNFα expression (Figure 3A). The expression of cleaved caspase-3 also decreased following VPA pretreatment in salicylate-induced cortical neurons (Figure 4B,D).

### 2.5. GPIAS 

**Figure 5:**
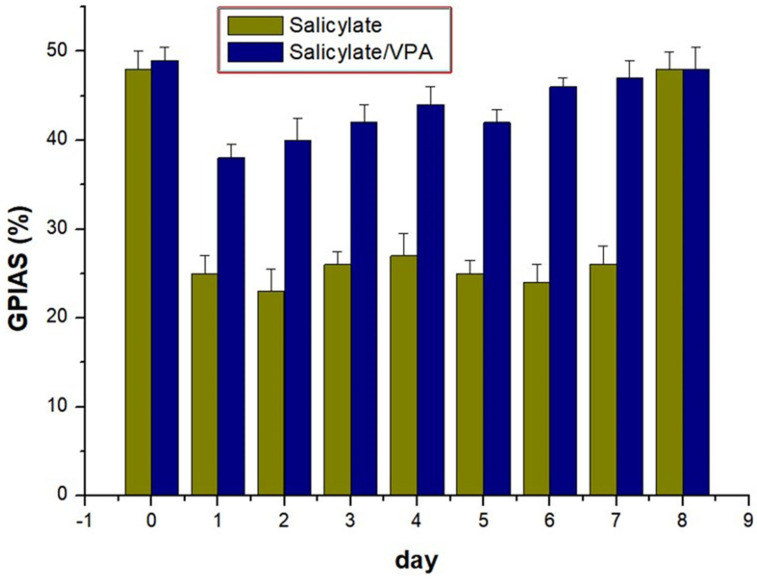
The salicylate/VPA group showed significantly attenuated GPIAS ratio compared with that in the salicylate group from days 1 to 7 (days 1–4, *p* < 0.05; days 5–7, *p* < 0.01).

During the baseline sessions, the mean GPIAS values were 48.3% in the control group and 49.2% in the study group. The mean GPIAS values decreased in the control group, indicating induction of tinnitus in the rats. In contrast, in the experimental group treated with both salicylate and VPA, the average GPIAS values were comparable to those of the control group, indicating that salicylate-induced tinnitus was improved by VPA treatment from days 1 to 7 (all *p* < 0.05) (Figure 5). 

### 2.6. Auditory Brainstem Response

**Figure 6:**
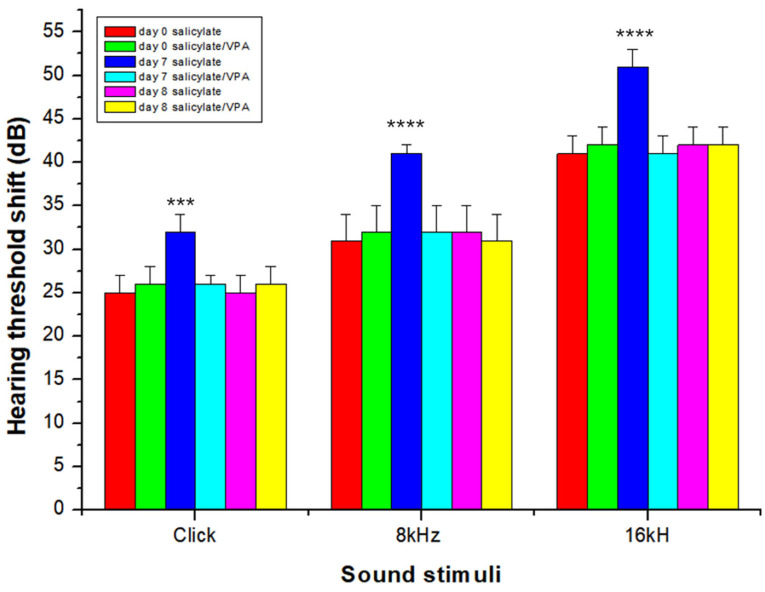
Statistically significant temporary ABR threshold shift was observed in the salicylate group on day 7 compared with ABR threshold shift before treatment (click, *** *p* < 0.001; 8 kHz, **** *p* < 0.0001; 16 kHz, **** *p* < 0.0001). One-way ANOVA followed by post hoc Tukey test using GraphPad Prism 8.0.

To determine whether salicylate could induce a temporary auditory threshold shift, ABR threshold shifts were observed before drug treatment and on days 7 and 8 (1 day after the end of treatment). Statistically significant temporary ABR threshold shift was observed in the salicylate group on day 7 compared with ABR threshold shift before treatment (click, *p* < 0.001; 8 kHz, *p* < 0.0001; 16 kHz, *p* < 0.0001). However, the salicylate/VPA group showed attenuation of the threshold shift on day 7. The mean ABR threshold in both the groups was not significant before and 1 day after the end of treatment (*p* = 0.15) (Figure 6). These results indicated that VPA attenuated salicylate-induced threshold shift. 

### 2.7. MicroPET CT Findings

**Figure 7:**
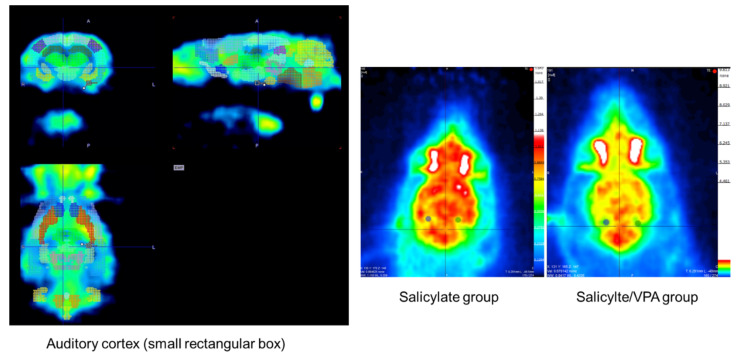
The average SUVs of each auditory cortex in the salicylate group were 0.91198 and 0.84824, whereas those in the salicylate/VPA group decreased to 0.48918 and 0.48345.

An image representing microPET scan is presented (Figure 7). The average SUVs of each auditory cortex in the salicylate group were 0.91198 and 0.84824, whereas those in the salicylate/VPA group were decreased to 0.48918 and 0.48345 (*p* < 0.05). 

### 2.8. Spatial Expression of NR2B in the Auditory Cortex after Salicylate and VPA Treatments 

**Figure 8:**
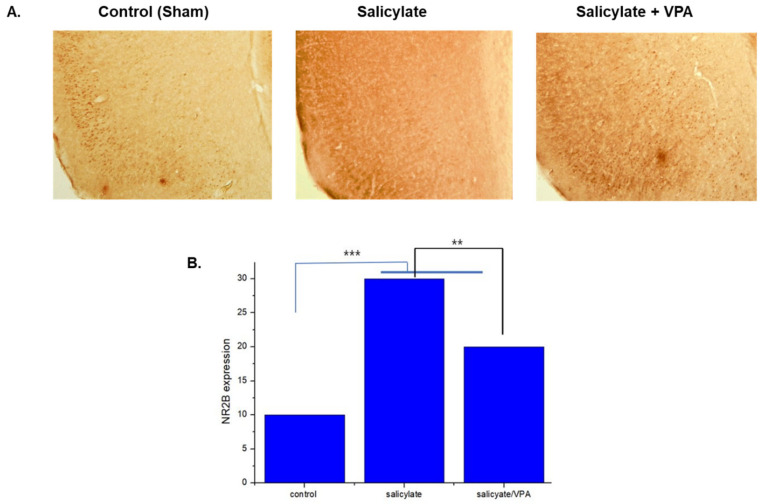
Immunolabeling of NR2B in the auditory cortex (**A**) and protein expression levels were quantified using Image J software (**B**) The salicylate group showed increased NR2B expression compared with that of the control, but VPA attenuated salicylate-induced increase in NR2B expression. ** *p* ≤ 0.01; *** *p* ≤ 0.001. One-way ANOVA followed by post hoc Tukey test using GraphPad Prism 8.0.

We examined the expression of NR2B in the auditory cortex of the rat brain using immunohistochemistry. Representative photomicrographs of NR2B expression in the auditory cortex are shown in Figure 8A. The expression of NR2B was increased significantly in the salicylate-treated group compared with that of the vehicle-treated control group (Figure 8B). In the group treated with salicylate/VPA, NR2B expression was decreased to a level comparable to that of the vehicle-treated group. 

## 3. Discussion

VPA is a short-chain fatty acid commonly used as an antiepileptic drug. The pharmacological action of VPA in the treatment of epilepsy involves multiple mechanisms, including those associated with the regulation of GABAergic neurotransmission. Previous studies have reported that VPA regulates the conversion of arachidonic acid through COX in the rat brain [14]. 

NMDA is a synthetic compound that selectively activates NMDA receptors, which are a subtype of glutamate receptors. The mode of action of NMDA is similar to that of glutamate. When glutamate receptors are excessively activated, it results in an acute rise in intracellular calcium levels and causes excitotoxicity. Effective inhibition of NMDA receptors may be a potential therapeutic strategy for tinnitus [15]. Once the neurons are stressed due to chronic glutamate excitotoxicity, they eventually undergo cell death [16]. In a previous study, treatment with salicylate was found to increase intracellular ROS levels in the spiral ganglion neurons of mice with tinnitus [17,18], which eventually killed auditory neuron cells through the intrinsic apoptosis pathway [19,20]. In our present study, endogenous ROS levels in neuronal cells were increased in the salicylate group and were subsequently reduced in the VPA-pretreated group. In addition, expression of the apoptotic protein, cleaved caspase-3, was observed to be reduced following pretreatment with VPA. 

VPA has been previously known to modulate NMDA receptor activity by down-regulating the expression of two NMDA receptor-interacting proteins, namely, postsynaptic density protein PSD-95 and CaMKII [21,22]. Thus, VPA relieves the spontaneous excitatory postsynaptic potential by controlling NMDA receptors. In the present study, attenuation of ABR threshold shift in the salicylate/VPA group may be due to the indirect regulation of NMDA receptors by VPA. 

Animals receiving long-term administration of salicylate develop tinnitus and hearing loss due to structural abnormalities in the central auditory system and frontal cortex [23]. Tinnitus is also caused by the abnormal activity of NMDA receptors in the synapses of inner hair cells [24]. We previously established a salicylate-induced tinnitus model, which was characterized by the expression of NMDA receptors and related genes [9,25,26]. In the present study, we investigated salicylate-induced tinnitus in both cell and animal models. We found that VPA exhibits greater beneficial effects in these models by preventing excessive activation of the NMDA receptor, as observed by the reduced expression of NR2B and related genes. 

The elevated levels of NMDA receptor due to administration of high doses of salicylate increases intracellular Ca^2+^ influx and induces excitotoxicity [15,27]. Neuronal hyperactivity by NMDA receptors could also stimulate plasticity signals, such as CREB phosphorylation [12,28]. VPA is known as an excitatory inhibitor of prefrontal cortical neurons and is widely used to treat bipolar disorder, epilepsy, and migraine headaches [29]. In the present study, when the cells were pretreated with VPA, the salicylate-induced increase in NR2B and CREB phosphorylation decreased. Thus, salicylate stimulated NR2B activation and increased CREB phosphorylation, which are associated with glutamate excitotoxicity. Furthermore, VPA may prevent excessive NMDA receptor activation and protect the hair cells and neurons from this damage. However, we did not examine the cochlear NMDA level since it has already been reported [30]. 

In brain imaging research, microPET can reflect neurobiochemistry or pharmacokinetics using various radiotracers. Recently, PET and functional magnetic resonance imaging have been used to study brain areas for visualizing brain activation. Moreover, ^18^F-FDG is a fluorine 18-labeled glucose analog that is transported into the cell, phosphorylated, and trapped within the cytoplasm. Changes in the rate of glucose metabolism probably correspond to changes in neurotransmitter function [31]. More recently, ^18^F-FDG microPET scan, a functional neuroimaging technique, has become available for visualizing brain activity by quantifying glucose consumption in rats. MicroPET imaging has been used to examine changes in glucose metabolism in rats after induction, such as behavioral challenges [32] and brain ischemia [33]. To date, there are no reports using microPET in rats with salicylate-induced tinnitus. In the present study, the SUVs were significantly decreased in the VPA-pretreated group compared with those in the salicylate group. In addition, NR2B expression was reduced in the VPA-pretreated group compared with that in the salicylate group. 

Regardless of the antioxidant properties of salicylate, high doses have been observed to cause an upregulation of superoxides, leading to neuronal cell death in the spiral ganglion in vitro. It has also been reported to interfere with the neurons in the central auditory system. This could be due to the influence of salicylate on γ-aminobutyric acid (GABA) in the CNS [34,35,36,37]. Moreover, studies have shown that the administration of drugs that increase various GABA-mediated inhibitory processes could suppress the hyperactivity in the auditory cortex induced by salicylate [38] (Figure S1). Although there are multiple mechanisms reported to be involved in the occurrence of tinnitus, we have focused on the excitatory action of salicylate, NMDA receptors, and VPA treatment, which is a limitation of the current study. We did not examine the molecular regulation of the inhibitory effects associated with GABA and VPA. Further studies could unravel more about these molecular mechanisms in tinnitus. To date, tinnitus generators are commonly found in many non-auditory high-dimensional treatment centers, as well as in and around the central, primary auditory cortex [39]. Recent tinnitus treatments that have been tried are as follows: (1) molecular level (intracochlear application of NMDA antagonist, modulation of microtubule-associated protein in molecular pathways, modulation of gamma-amino-butyric acid), (2) systemic level (transcranial magnetic stimulation, transcranial direct current stimulation, and vagus nerve stimulation [40]). VPA plays a role in decreasing NMDA activation and increasing the activation of gamma- aminobutyric acid.

## 4. Materials and Methods

### 4.1. Cell Culture and Treatment

SH-SY5Y cells were grown in Dulbecco’s modified Eagle’s medium (DMEM)/F12 supplemented with 10% fetal bovine serum (FBS) at 37 °C in 5% CO_2_. Cells were harvested using 0.25% trypsin-EDTA solution. Then, 1.5 × 10^6^ cells were reseeded in a 100-mm culture dish containing DMEM and 10% FBS. For differentiation into neuron-like cells, cells were cultured in a differentiation media formulated with 0.1% FBS, 1% Pen Strep, and 1 μM retinoic acid (Sigma, St. Louis, MO, USA). The cells were further incubated for 2 days. 

On the 18th embryonic day, the cerebral cortex of Sprague–Dawley rat pups was dissected and prepared for cultivation. Papain dissociation buffer (10 units/mL; Worthington Biochemical, Lakewood, NJ, USA) was used for tissue digestion along with 100 units/mL of DNase I (Roche, Basel, Switzerland). The tissues were digested for 30 min at 37 °C. Once digested, the tissues were further lysed into a single-cell suspension with the Neurobasal A medium (Invitrogen, Grand Island, NY, USA). The cells were seeded over poly-d-lysine hydrobromide (150 μg/mL; Sigma, St. Louis, MO, USA)-coated 12-well plates at a density of 0.5 × 10^6^ cells/well. One hour after plating, the seeding medium was replaced with a growth medium composed of Neurobasal A, 1 × B27 supplement (Thermo Fisher Scientific; Waltham, MA, USA), 100 units/mL penicillin, 0.1 mg/mL streptomycin, and 0.5 mM glutamine (Thermo Fisher Scientific; Waltham, MA, USA). Cultures were then incubated at 37 °C in 5% CO_2_.

Both SH-SY5Y cells (0.1% serum medium) and rat cortical neurons were treated with 200 µg/mL VPA for 12 h in serum and subsequently treated with 40 µg/mL salicylate for 8 h. 

### 4.2. Quantitative Polymerase Chain Reaction (qPCR)

Total RNA was extracted from SH-SY5Y cells using RNAiso reagent (Takara, Tokyo, Japan). cDNA was synthesized using the Primescript II 1st strand cDNA synthesis kit (Takara). For reverse transcription, 2 µg total RNA with 5 µM random primers, 1 mM of each dNTP, and the supplied buffer were used. qPCR was performed using the synthesized cDNA using Power SYBR Green PCR master mix (Applied Biosystems, Inc., Foster City, CA, USA) with primers for *NR2B*, *ARC*, and *TNFα*. The real-time PCR (StepOnePlus Real-Time PCR System) conditions were as follows: denaturation at 95 °C for 10 min, followed by 40 cycles of 15 s of extension and annealing at 95 °C, and 1 min at 60 °C. The primers were synthesized at GenoTech Corp. (Daejeon, Korea) and Integrated DNA Technologies, Inc., (Coralville, IA, USA) and are summarized in Supplemental Table S1.

### 4.3. Immunoblot Analysis

Total proteins were isolated from the cells using 50 µL RIPA buffer (Santa Cruz Biotechnology, Santa Cruz, CA, USA) with the addition of inhibitor cocktail and phenylmethylsulfonyl fluoride for 30 min on ice and then centrifuged at 16,000× *g* for 20 min. The protein concentration was measured using a BSA assay kit (Thermo Fisher Scientific; Waltham, MA, USA). Using the isolated proteins, an immunoblot assay was performed using appropriate primary antibodies against NMDAε2 (1:200), p53 (1:200), CREB (1:500), p-CREB (1:500), GAPDH (1:1000) (Santa Cruz Biotechnology), and caspase-3, active (cleaved) form (1:150) (Merck Millipore, Temecula, CA, USA) and incubated at 4 °C overnight. Thereafter, horseradish peroxidase-conjugated anti-goat, anti-rabbit, and anti-rabbit secondary antibodies were used (Santa Cruz Biotechnology). Protein bands were captured on X-ray films using the GE ECL detection system. 

### 4.4. Immunocytochemical Staining

SH-SY5Y cells were cultured over poly-L-lysine-coated (150 μg/mL; Sigma-Aldrich) coverslips and differentiated into neuron-like cells. After treatments, cells were fixed using 4% paraformaldehyde for 15 min and permeabilized using methanol for 5 min. Cells were incubated with antibodies against NMDAε2 and p-CREB (1:200; Santa Cruz Biotechnology) in phosphate-buffered saline (PBS) for 1.5 h at room temperature (RT), and then incubated with Alexa 555-conjugated donkey anti-mouse IgG and Alexa 488-conjugated donkey anti-rabbit IgG secondary antibodies (1:500; Thermo Fisher Scientific) in PBS along with Hoechst 33,342 (1:1000; Thermo Fisher Scientific) for 1.5 h at RT. The cells were then washed thrice with PBS, mounted over a glass slide using the ProLong Gold anti-fade reagent (Molecular Probes Inc., Eugene, OR, USA), and visualized under a Nikon Eclipse Ti2 fluorescence microscope (Nikon, Tokyo, Japan). Images were captured using a DS-Ri2 digital camera (Nikon). 

### 4.5. Detection of Intracellular ROS 

Once the SH-SY5Y cells were treated with the drugs, intracellular ROS levels were measured using 2′,7′-dichlorofluorescein diacetate (DCFH-DA), which is converted to the fluorescent byproduct, 2′,7′-dichlorodihydrofluorescein. Cells were cultured in a 24-well plate; then, 20 µM DCFH-DA diluted in serum medium was added before cells were incubated at 37 °C for 20 min. After washing with PBS, cells were observed under a Nikon Eclipse Ti2 fluorescence microscope and photographed by a DS-Ri2 digital camera. 

### 4.6. In Vivo Experiments

#### 4.6.1. Animals

**Figure 9:**
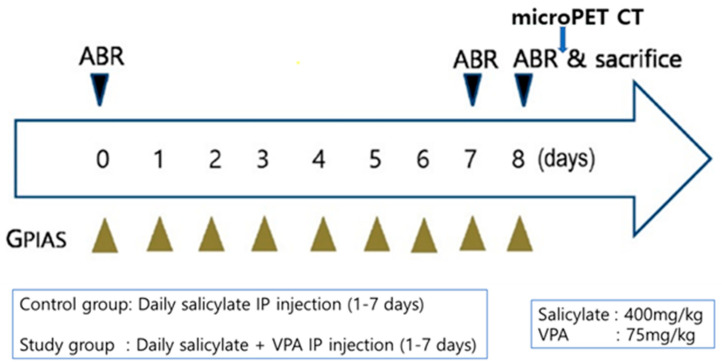
Flow chart of the in vivo study.

Experiments were designed using 20 adult male Sprague–Dawley rats (weighing 300 g) with normal eardrums. All procedures were approved by the Institutional Animal Care and Use Committee (C IACUC-2020-S0019). The animals were randomly divided into either a control group (*n* = 5) or a study group (*n* = 5) for GPIAS evaluations. The control group was used to evaluate salicylate-induced tinnitus, whereas the study group was used to assess the therapeutic effect of VPA on salicylate-induced tinnitus. The control group was injected intraperitoneally (IP) with 400 mg·kg^−1^·day^−1^ of sodium salicylate (Sigma), whereas the study group was injected IP with sodium salicylate combined with 75 mg·kg^−1^·day^−1^ of VPA (Sigma) for 7 consecutive days (Figure 9).

#### 4.6.2. Gap and Noise Burst Prepulse Inhibition of Acoustic Startle

To obtain the GPIAS value, a startle response measurement system was used as described in our previous study [9]. In brief, the system comprised a mesh cage with a vibration sensor, noise box with an anechoic inner wall, acoustic stimulator (PM-5004 amplifier; Marantz, Kawasaki, Japan, with a full-range loud speaker), reference microphone (40 PH; GRAS, Holte, Denmark), sensor signal acquisition hardware (PC and NI PCIe-6321; National Instruments, Austin, TX, USA), and LabVIEW-based custom graphical user interface (GUI) software. To detect the startling vibration in rats, an accelerometer sensor module was attached to the bottom plate of the mesh cage. The processes to acquire the startle response via acoustic stimulation were controlled by our implemented GUI software, which also performed the analysis process for obtaining the GPIAS values. An integrated system within the soundproof box was used to obtain all startle response measurements. To measure GPIAS, every rat underwent a session comprising 15 gap-conditioned stimuli-evoked startle responses and 15 responses with no gap. In addition to these, two types of acoustic stimuli (gap and no gap) were performed on rats in a random order. The rats were placed in the cage before the start of the first session for approximately 2 min to acclimatize them to the measurement environment. A narrow-band background noise with 1 kHz, 16 kHz, and 60 dB of bandwidth, center frequency, and sound pressure level (SPL), respectively, along with a short high-level stimulus (50 ms length, onset at 100 ms) prior to the onset of startle (stimulus) for startling were used for acoustic stimulation for GPIAS. A prepulse gap of 50 ms length, onset of 100 ms in advance of the onset of the startle stimulus, was inserted ahead of the startle stimulus procedure. 

#### 4.6.3. Measurement of Auditory Brainstem Response

Hearing function was evaluated using an ABR measuring system (Tucker-Davis Technologies, Miami, FL, USA). Before beginning drug treatments (day 0), ABR thresholds of all animals were obtained. Once the drug treatment periods were complete on days 7 and 8, ABR measurements were acquired according to the previously reported procedure [9]. The subdermal needle electrodes were placed at the vertex, and the reference electrode was inserted at the occiput. The hearing threshold was evaluated by using tone bursts (2 ms rise/decay, 1 ms plateau) in a decreasing intensity at a rate of 50/s, starting with the levels that stimulated unique evoked potentials. Furthermore, 90 dB SPL was used as the starting stimulus, and the process was repeated at a decrease of 10 dB for every complete recording until no response could be detected. 

#### 4.6.4. ^18^F-FDG PET Imaging

Rats were maintained using 2% isoflurane/oxygen anesthesia. The PET brain images were acquired using a preclinical PET-CT scanner (SuperArgus 4R, Sedecal, Madrid, Spain). The animals were fasted for 6 h prior to PET scans. The static PET data were acquired at 30 min after tail vein injection of ^18^F-FDG (14.8 MBq). After the 20 min of PET scan, a CT study was acquired for attenuation correction and anatomical co-registration. The volumes of interest were drawn in the target area of the brain. The maximal and mean standardized uptake values (SUVs) were measured on the reconstructed images using the AMID v1.0.4 software. 

#### 4.6.5. Preparation of Free-Floating Sections

Rats were sacrificed 7 days after the injection of each drug or vehicle for histological examination of the brain tissue. The animals were anesthetized and perfused with 4% (*w*/*v*) paraformaldehyde (PFA) in PBS (pH 7.4). The brains were removed immediately, stored in 4% (*w*/*v*) PFA in PBS for 2 days at 4 °C, suspended in 30% (*w*/*v*) sucrose for 4 days, and then embedded in optimum cutting temperature compound (Miles Inc., Elkhart, IN, USA). Using a sliding microtome (SM2010R; Leica Microsystems, Wetzlar, Germany), the hemispheres were coronally sectioned at approximately 4.30–5.30 mm caudal to the bregma for the auditory cortex. Free-floating serial sections (30-μm-thick) were collected into 10 wells filled with PBS.

#### 4.6.6. Immunohistochemistry of Free-Floating Sections

Brain samples were collected and fixed using 4% (*w*/*v*) PFA in PBS. Samples were then immersed in 30% (*w*/*v*) sucrose for at least 4 days. Then, they were sectioned into coronal slices (30 μm) using a frozen sliding microtome (SM2010R) and stored in PBS at 4 °C. To disable intrinsic peroxidase activity, sections were incubated in 0.3% (*v*/*v*) hydrogen peroxide in distilled water for 20 min before subsequent blocking for 1 h with 5% (*v*/*v*) normal goat serum (Vector ABC Elite Kit; Vector Laboratories, Burlingame, CA, USA) in 0.3% (*v*/*v*) Triton X-100. Sections were incubated with rabbit anti-NMDAR2B (1:200; Abcam, Cambridge, UK) diluted with antibody dilution buffer (Invitrogen) at 4 °C overnight. After washing, sections were reacted with biotinylated goat anti-rabbit IgG (Vector ABC Elite Kit; Vector Laboratories) for 1 h and rinsed again. Then, sections were incubated for 1 h at RT with avidin-biotin peroxidase complex (Vector ABC Elite Kit; Vector Laboratories) following the manufacturer’s instructions. After washing, a diaminobenzidine substrate (DAB kit; Vector Laboratories) was used for the peroxidase reaction. 

The immunoreactivity of NMDAR2B in the auditory cortex subregions was analyzed by measuring the intensity of the NMDAR2B-immunopositive reaction with the ImageJ software. Coronal sections (30 μm thick) were selected at approximately 2.5–3.6 mm posterior to the bregma in each brain, and the intensities in the subregion were assessed. The levels of intensity were expressed as means ± standard errors (SEs; *n* = 4 rats/group).

### 4.7. Statistical Analyses 

Data are represented as the mean ± standard deviation (SD) of three or more independent experiments. Statistical comparisons between groups were made using one-way ANOVA followed by post hoc Tukey test using GraphPad Prism 8.0. A *p* value < 0.05 was considered statistically significant. In the in vivo test, ABR thresholds and GPIAS were compared between the control (salicylate) and study group (salicylate/VPA) at each time point using t test. Expression levels of NR2B as quantified by image analysis were compared by one-way ANOVA followed by post hoc Tukey test.

## 5. Conclusions

In the present study, our results revealed that VPA attenuated salicylate-induced auditory dysfunction. 

## Data Availability

The data presented in this study are available on request from the corresponding author.

## Supplementary Materials

The following are available online at https://www.mdpi.com/article/10.3390/ijms23010023/s1.
