# Supplementary material for: Neuroprotective Effect of Valproic Acid on Salicylate-Induced Tinnitus
Source: Int J Mol Sci. 2021 Dec 21;23(1):23. doi: 10.3390/ijms23010023 (PMC8744959; doi:10.3390/ijms23010023)
Supplement: Supplementary file 1 [file ijms-23-00023-s001.zip › ijms-1480550-supplementary.pdf]

International Journal of
Molecular Sciences

MDPI

Supplementary materials

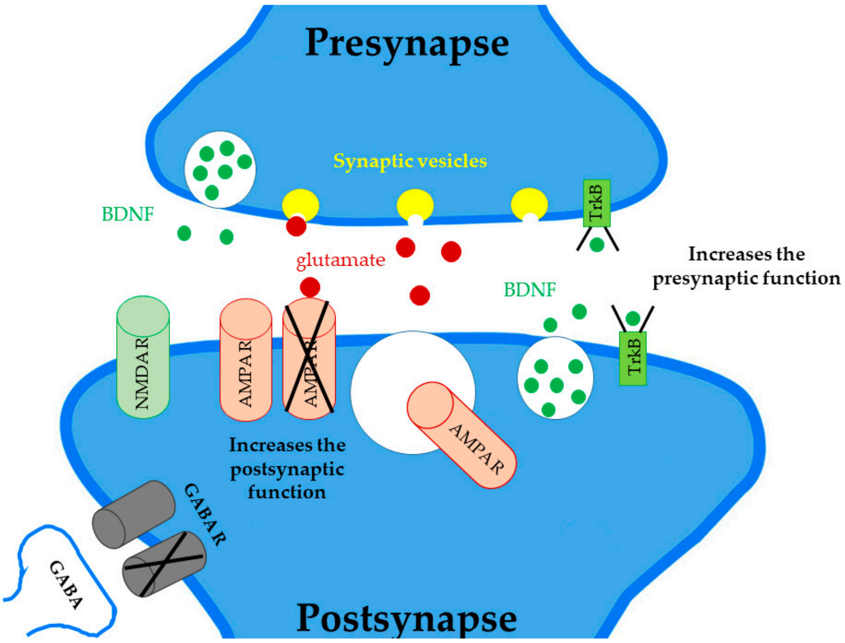

Figure S1. Shows the molecular mechanism behind the influence of salicylate treatment on GABA.

Table S1. Oligonucleotides sequences used for the quantitative polymerase chain reaction.

Gene	Forward primer (5'→3')	Reverse primer (3'→5')	Gene accession
Human β-actin	ATC CGC AAA GAC CTG TAC GC	TCT TCA TTG TGC TGG GTG CC	NM_001101
Human NMDA(NR2B)	GGAGAGGTGGTCATGAAGAG	CATTGCTGCGTGACACCATG	NM_000834.4
Human TNFα	GTTGTAGCAAACCCTCAAGCTG	CCAGCTGGTTATCTCTCAGCTC	NM_000594.3
Human ARC	ACAACAGGTCTCAAGGTTCCC	AGCCGACTCCTCTCTGTAGC	NM_015193.4
Rat GAPDH	CTGCCACTCAGAAGACTGTGG	TTCAGCTCTGGGATGACCTTG	NM_017008.4
Rat NMDA (NR2B)	GGAGATGGAAGAACTGGAAGCTC	GACACCTGCCATATTGTCGATG	NM_012574.1
Rat TNFα	CCACCACGCTCTTCTGTCTAC	GATGATCTGAGTGTGAGGGTCTG	NM_012675.3
Rat ARC	GTCTGCTGCATAGAAGGAACCAG	AGGGTGCCCACCACATACTGA	NM_019361.1
